# Telehealth and Physical Therapy Clinical Decision Making in a Patient with a Falcine Meningioma

**DOI:** 10.5195/ijt.2020.6302

**Published:** 2020-06-30

**Authors:** Ryan Boggs, Nicholas Frappa, Michael Ross, Michael Tall

**Affiliations:** 1 Department of Physical Therapy, Daemen College, Amherst, NY, USA; 2 Department of Radiology, University of Texas Health Science Center at San Antonio, San Antonio, TX, USA

**Keywords:** Medical screening, Physical therapy, Telehealth

## Abstract

Telehealth utilizes information technologies and communication networks to deliver healthcare and education with lower costs and improved access, quality, and efficiency of healthcare services. This report describes the application of telehealth for medical screening, clinical decision making, and medical referral in a physical therapy practice. The patient described was a 50-year old man who contacted his physical therapist via telephone for a chief complaint of worsening left sided numbness and tingling that began insidiously 2 days prior. Further questioning revealed that the patient also complained of left ankle weakness, and slight unsteadiness with walking. He had not been feeling well and had been experiencing increasing bouts of unexplained fatigue over the previous two months that were now interfering with his work and recreational activities. The patient was evaluated by his physician the next day. Magnetic resonance imaging of the brain revealed a large (4 cm) falcine meningioma in the right parietal region. The patient was immediately referred to a neurosurgeon and underwent a craniotomy and tumor resection ten days later and subsequent gamma knife radiosurgery of the residual tumor bed two months after craniotomy and tumor resection. Follow-up imaging one year later revealed no evidence of recurrence or residual tumor. This patient case underscores the importance of recognizing signs and symptoms of serious disease, and how referral following telehealth via telephone can inform diagnosis.

Telehealth is defined by the American Physical Therapy Association as the use of electronic communication to remotely provide health care information and services (American Physical Therapy Association, 2019). Telehealth, which may consist of telephone-based communication (though more typically employs an audio-visual connection) utilizes information technologies and communication networks for delivering healthcare and education. Telehealth use can lower costs as well as improve access, quality, and efficiency of healthcare services. Access to telehealth comes at a critical time for patients who are seeking treatment in the present complex healthcare system, especially during a Coronavirus (COVID-19) pandemic.

With an expected shortage of physicians in excess of 100,000 within the next decade and overcrowding of many emergency departments, mortality rates are increased, treatments are delayed for time-sensitive conditions, and patient satisfaction is drastically reduced (Basu et al., 2019; Derlet, 2002; Di Somma et al., 2014; Prentice & Pizer, 2007; Sonis et al., 2018; Trzeciak & Rivers, 2003; Yarmohammadian et al., 2017). To address the problems of decreased access to primary care providers, especially in rural areas, physical therapists have emerged as extended scope practitioners. In this role they provide triage services and help provide other services to patients with musculoskeletal disorders. It is well documented that the use of physical therapists as extended scope practitioners results in decreased wait times for care. Furthermore, there is no difference in the clinical diagnostic accuracy of physical therapists and physicians in an orthopedic setting, and no reported adverse events when patients were seen by physical therapists first in a direct access mode. Physical therapists were able to independently and safely manage a caseload in both routine outpatient settings and emergency departments, as well as to successfully identify conditions needed for referral (Bird et al., 2016; De Grunchy et al., 2015; Moore et al., 2005; Razmjou, 2013; Sutton et al., 2015).

The use of telehealth by physical therapists is becoming more frequent across the United States in both the military and civilian settings for musculoskeletal triage and consultation (Lee et al., 2018). Additionally, studies have reported on the efficacy of telehealth in terms of outcome and cost in the delivery of rehabilitation services (Grona et al., 2018; Lee & Harada, 2012; Nelson et al., 2017). Yet, the widespread adoption of telehealth in physical therapy practice has been stalled by concerns about implementation, policy barriers, and billing for services (Lee & Harada, 2012).

The purpose of this report is to describe the use of telehealth for medical screening, clinical decision making, and medical referral for a patient who contacted his physical therapist via telephone for a chief complaint of left sided paresthesias. The patient was subsequently diagnosed with a large right paracentral falcine meningioma.

## CASE REPORT

**Figure 1.**
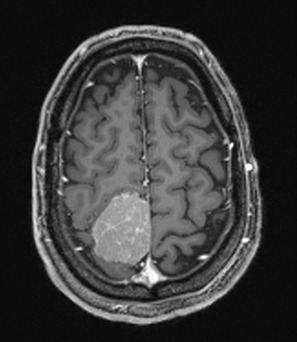
Axial view magnetic resonance image of the brain.

*Note*. Figure shows a 4 cm in diameter right parafalcine mass which showed heterogeneous enhancement and mass effect on the right parietal lobe. This was characteristic of a meningioma.

The patient was a 50-year old man (body mass index = 29.8 kg/m^2^) who contacted his physical therapist via telephone for a chief complaint of worsening left sided numbness and tingling. The patient owned his own company and was quite active. The patient's symptoms began insidiously two days prior and he had not contacted his physician about his concern. The patient reported that when he awoke in the morning, his left arm and leg were tingling and felt heavy. Further questioning revealed that the patient also complained of left ankle weakness, and slight unsteadiness with walking. Also, he had not been feeling well and had been experiencing increasing bouts of unexplained fatigue over the previous two months that were now interfering with his work and recreational activities. The patient denied any right sided symptoms, facial paralysis, dizziness, dysphagia, dysarthria, drop attacks, diplopia, weight loss, or problems with bowel or bladder function. The patient's past medical history was unremarkable and he denied any medication use. He had quit smoking 24 years ago. His family history revealed that his mother had succumbed to a malignant gliobastoma at age 45 and his father had been diagnosed with colon cancer at age 65. Due to the concern that the patient's presentation was consistent with possible sinister pathology, the physical therapist referred the patient to his primary care physician for an immediate evaluation. The patient was evaluated by his physician the next day. Magnetic resonance imaging of the brain revealed a large (4 cm) parafalcine mass that was characteristic of a meningioma in the right parietal region (Figure 1).

The patient was immediately referred to a neurosurgeon and underwent a craniotomy and tumor resection 10 days later; over the course of those 10 days, the patient also experienced seizure like activity on two separate occasions. Post-surgical pathology reports indicated an increased MIB-1 index which is concerning for increased risk of recurrence (Abry et al., 2010; Li et al., 2019). This resulted in an oncology referral and subsequent gamma knife radiosurgery of the residual tumor bed 2 months after craniotomy and tumor resection. Follow up imaging 1 year later revealed no evidence of recurrence or residual tumor. The patient has subsequently returned to his previous level of function with resolution of all symptoms except for some residual slight ankle dorsiflexion weakness that never fully resolved.

## DISCUSSION

An important role for physical therapists in the healthcare delivery system is to recognize when patient referral to a physician or other healthcare provider is indicated. The patient described in this report contacted his physical therapist via telephone for a consultation due to a 2-day history of worsening left sided numbness and tingling. Due to the concern that the patient's presentation was consistent with possible sinister pathology, the physical therapist referred the patient to his primary care physician for an immediate evaluation and he was subsequently diagnosed with a large (4 cm) right falcine meningioma in the parietal region. While several published patient case reports have described in-clinic physical therapist/patient episodes of care that resulted in the referral of the patient to a physician and a subsequent diagnosis of medical disease, we are unaware of any such cases that have been done primarily through the application of telehealth measures (Boissonnault & Ross, 2012). This patient case report may provide some insight for physical therapists and other providers regarding how patients with occult medical disease may access physical therapist services through telehealth, how these conditions may present during a physical therapy telehealth encounter, and the physical therapist's role in the diagnostic and referral process. It should be noted that a video-based telehealth connection would provide even more information (e.g., appearance, facial and body movement) than an audio-only telephone connection, and that most telehealth sessions are currently conducted in video-audio mode.

In a review of 78 patients that a physical therapist had referred to a physician and resulted in a subsequent diagnosis of medical disease, the patients' primary presenting symptoms included pain (n = 60), weakness (n = 4), tingling/numbness (n = 2), or a combination (n = 12) (Boissonnault & Ross, 2012). The patient in this case study's primary complaint of left sided numbness and tingling is one of the less common symptom profiles that are seen in outpatient ambulatory settings by physical therapists that requires medical referral. There are several causes of paresthesia including peripheral neuromusculoskeletal and central nervous system pathology, as well as systemic disease (Berkowitz, 2017) While there may be diagnostic uncertainly regarding the etiology of paresthesia as many different disorders may have a similar symptom profile, other signs and symptoms (e.g., entire limb or hemibody paresthesia, weakness especially if more than one myotome is affected, unsteadiness with gait, changes in general health such as fatigue or malaise) would help the provider determine if there is concern over sinister pathology and if diagnostic imaging or medical referral is warranted through the application of telehealth. It is important for physical therapists to be familiar with differential diagnosis, including screening for serious underlying pathology and making the appropriate referral when necessary (Berkowitz, 2017).

Typically, if unilateral and located to one extremity, a peripheral nerve entrapment, radiculopathy, or plexus lesion may be implicated (Dumitru et al., 2002; Preston & Shapiro, 2013). However, the involvement of unilateral upper and lower extremities with no clear dermatomal or peripheral nerve distribution is suggestive of a possible spinal cord or brain lesion (Berkowitz, 2017). Given the symptoms of the patient in this case and the subsequent concern of a central nervous system lesion, the patient was immediately referred to his primary care physician for imaging. Brain tumors generally cause the development of focal neurologic deficits including hemiparesis and sensory deficits (Mukand et al., 2001). However, benign tumors, such as meningiomas, may not produce any symptoms until they have enlarged significantly (Marta et al., 2011).

Meningiomas account for approximately 30% of all primary brain and central nervous system tumors reported in the United States (Baldi et al., 2018). Women are more than twice as likely to develop meningiomas which is attributed to sex hormones and/or genetic disposition (Baldi et al., 2018). Interestingly, an increased risk of primary brain tumors exists among relatives of glioma patients (Wrensch et al., 1997). This risk factor was noted in the patient's past medical history and contributed to the clinical decision that the symptoms warranted an immediate referral. Once suspected, surgical excision of the tumor is required to confirm the diagnosis and achieve the complete resection of the tumor. Since elevated MIB-1 is predictive of the regrowth potential of a tumor after initial surgery, additional referral to oncology with subsequent intervention was performed (Abry et al., 2010; Li et al., 2019).

## CONCLUSION

Appropriate and prudent practitioner screening and referral is necessary if clinical symptoms suggest a condition that requires the services of another provider. In this case, the prudent use of telehealth and an understanding of the clinical findings associated with sinister pathology enabled the physical therapist to refer the patient to his primary care physician for an immediate evaluation. Prompt diagnosis of the patient's condition was made and primary treatment was initiated in a timely fashion.

This patient case underscores the contribution that physical therapists practicing via telehealth can make by discerning the signs and symptoms of serious disease and making appropriate referrals. It should be noted that while evidence is growing that demonstrates the clinical utility of telehealth, there are circumstances in which a remote examination may not entirely replace a comprehensive history and physical examination conducted in an in-person clinical setting.

## AUTHORS' NOTE

We declare that the patient has granted the authors written permission to report his case in this manuscript. Ethics review from our institutional Human Subjects Research Review Committee was not required for this retrospective single subject case report.
